# Supplementary figures and images for: Satellite monitoring of terrestrial plastic waste
Source: PLoS One. 2023 Jan 18;18(1):e0278997. doi: 10.1371/journal.pone.0278997 (PMC9847976; doi:10.1371/journal.pone.0278997)

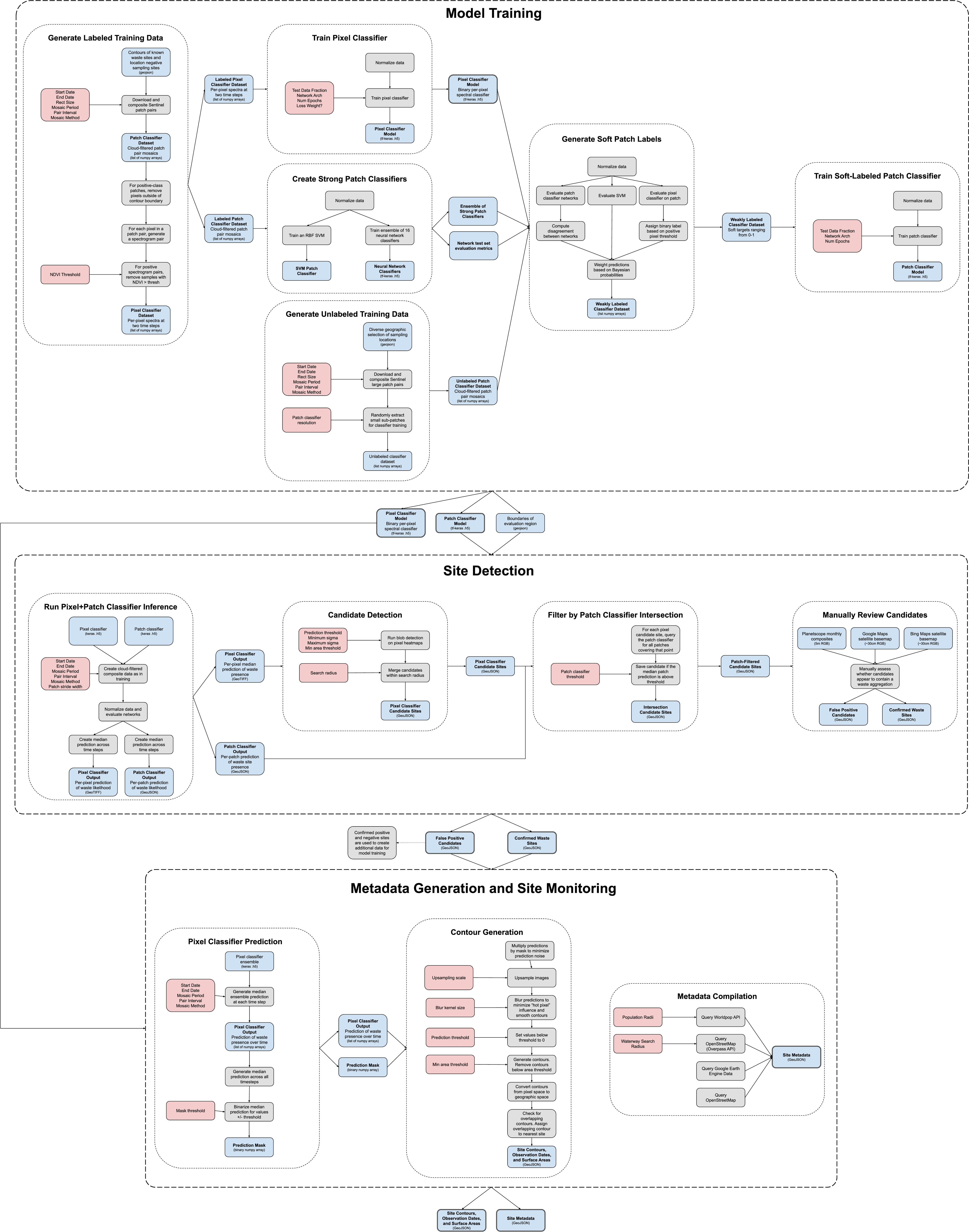

Supplement: S1 Fig — Diagram showing the methodology pipeline in greater detail. Elements are colored according to type. Processing stages are shown in gray, processing configuration parameters in red, and outputs in blue. Major pipeline components grouped and contained within dashed outlines. (TIF) [file pone.0278997.s001.tif]
